# A multiscale approach to mapping seabed sediments

**DOI:** 10.1371/journal.pone.0193647

**Published:** 2018-02-28

**Authors:** Benjamin Misiuk, Vincent Lecours, Trevor Bell

**Affiliations:** 1 Department of Geography, Memorial University of Newfoundland, St. John’s, Newfoundland, Canada; 2 Department of Fisheries and Aquatic Sciences, School of Forest Resources and Conservation, University of Florida, Gainesville, Florida, United States of America; Centro de Investigacion Cientifica y de Educacion Superior de Ensenada Division de Fisica Aplicada, MEXICO

## Abstract

Benthic habitat maps, including maps of seabed sediments, have become critical spatial-decision support tools for marine ecological management and conservation. Despite the increasing recognition that environmental variables should be considered at multiple spatial scales, variables used in habitat mapping are often implemented at a single scale. The objective of this study was to evaluate the potential for using environmental variables at multiple scales for modelling and mapping seabed sediments. Sixteen environmental variables were derived from multibeam echosounder data collected near Qikiqtarjuaq, Nunavut, Canada at eight spatial scales ranging from 5 to 275 m, and were tested as predictor variables for modelling seabed sediment distributions. Using grain size data obtained from grab samples, we tested which scales of each predictor variable contributed most to sediment models. Results showed that the default scale was often not the best. Out of 129 potential scale-dependent variables, 11 were selected to model the additive log-ratio of mud and sand at five different scales, and 15 were selected to model the additive log-ratio of gravel and sand, also at five different scales. Boosted Regression Tree models that explained between 46.4 and 56.3% of statistical deviance produced multiscale predictions of mud, sand, and gravel that were correlated with cross-validated test data (Spearman’s *ρ*_*mud*_ = 0.77, *ρ*_*sand*_ = 0.71, *ρ*_*gravel*_ = 0.58). Predictions of individual size fractions were classified to produce a map of seabed sediments that is useful for marine spatial planning. Based on the scale-dependence of variables in this study, we concluded that spatial scale consideration is at least as important as variable selection in seabed mapping.

## Introduction

Marine ecosystems provide a broad range of services to humans, including food, extractive resources, and cultural identity [1,2]. These systems are now being threatened and profoundly impacted on local and global scales by a suite of anthropogenic stressors such as climate change, overfishing, and pollution [3,4]. As pressures on marine systems intensify, there is an urgent need to monitor and mitigate impacts to ensure ecosystem viability and sustainable ecosystem services. Despite the importance of marine ecosystems to human well-being, and the immediate threats they face, we often lack the necessary information to make informed management decisions.

Seabed maps provide necessary information for a number of conservation and management applications. Habitat maps in particular are used to monitor anthropogenic impacts, to support government marine spatial planning, for marine protected area design, to generate knowledge about ecosystems and geology, and to assess seabed resources for economic and management purposes [5,6]. Benthic habitat mapping is defined broadly as “the use of spatially continuous data sets to represent and predict biological patterns on the seafloor” [7]. Habitat mapping can be applied to species, communities, or physical features of interest, but a fundamental requirement for generating useful maps in all cases is the availability of the appropriate high quality environmental spatial data.

Though benthic habitats are determined by a range of environmental variables, McArthur et al. [8] identified seabed substrate characteristics as the strongest independent predictors of benthic habitats. Sediment grain size is a particularly important substrate characteristic that can constrain the distribution of benthic habitats [9,10]. Along with other habitat-defining parameters, distribution maps of sediment grain size can thus serve as management tools for predicting the distribution of individual species and assemblages [11,12]. The increasing availability of accurate marine spatial data has improved our ability to map the distribution of seabed sediments. For instance, primary data collected from multibeam echosounders (MBES)–bathymetry (i.e. water depth) and backscatter (i.e. acoustic reflectivity)–and their derivatives can be used to delineate and model sediment grain size over large areas (thousands of km) at a high spatial resolution (metres) when coupled with ground-truth substrate samples (e.g. [12–14]). Quantitative predictions of sediment grain size can be used on their own as continuous explanatory variables in further analyses or can be classified for interpretation or use as categorical variables [15]. This quantitative predictive approach represents a departure from subjective expert-based interpretation towards more objective, repeatable methods [15,16].

Recent biological and geological modeling approaches have relied heavily on bathymetry-derived terrain variables (e.g. slope and rugosity) and backscatter-derived variables (e.g. hardness and heterogeneity) to predict the response of organisms (e.g. [17,18]), habitats (e.g. [19–21]), or sediment properties such as grain size or the presence of rock (e.g. [13,22]). Terrain variables can act as surrogates for patterns and processes on the seabed (e.g. seabed morphology, current dynamics, relative position) that may influence the distribution of sediments or biota [23]. While these processes are scale dependent (e.g. [24]), terrain variables are most often derived at the resolution of the primary data layers (bathymetry and backscatter) by default. The resolution of the primary data is selected by the data analyst, who must consider the specifications of the MBES system, the specific survey, and the quality of the data. Terrain variables are usually raster data products that are calculated using “focal” or “neighborhood” cell analyses on the primary layers. Deriving terrain variables at the resolution of the primary data imposes a spatial scale on them that may not be appropriate for representing the processes of interest [25–27]. To avoid the arbitrary selection of data scale it may be desirable to test at which scales explanatory variables have the greatest influence on the response variable. A solution that has been proposed in recent years is to move towards multiple scale or multiscale analyses [28]. *Multiple scale* analyses are those that consider data at multiple successive scales, and *multiscale* analyses are those that integrate information from multiple scales simultaneously [26]. Several terrestrial (e.g. [29–31]) and marine (e.g. [32–34]) studies have demonstrated that the use of data at different scales can affect results and interpretations. Since different environmental processes operate at different spatial scales [35], the adoption of a multiscale approach ensures that the relevant scale-dependent patterns and processes are captured [28].

The overall objective of this study was to evaluate the potential of multiscale approaches for predicting the distribution of sediment grain size for use in habitat mapping and marine spatial planning. Using a case study approach, we first examined whether using the default data resolution of terrain variables was optimal for predicting the distribution of sediment grain size. We then determined which terrain variables at which spatial scales most strongly influenced the distribution of sediment grain size. Finally, we applied this knowledge to predict distributions of mud, sand, and gravel fractions at optimal spatial scales in the study area.

## Data and methods

### Setting

**Fig 1:**
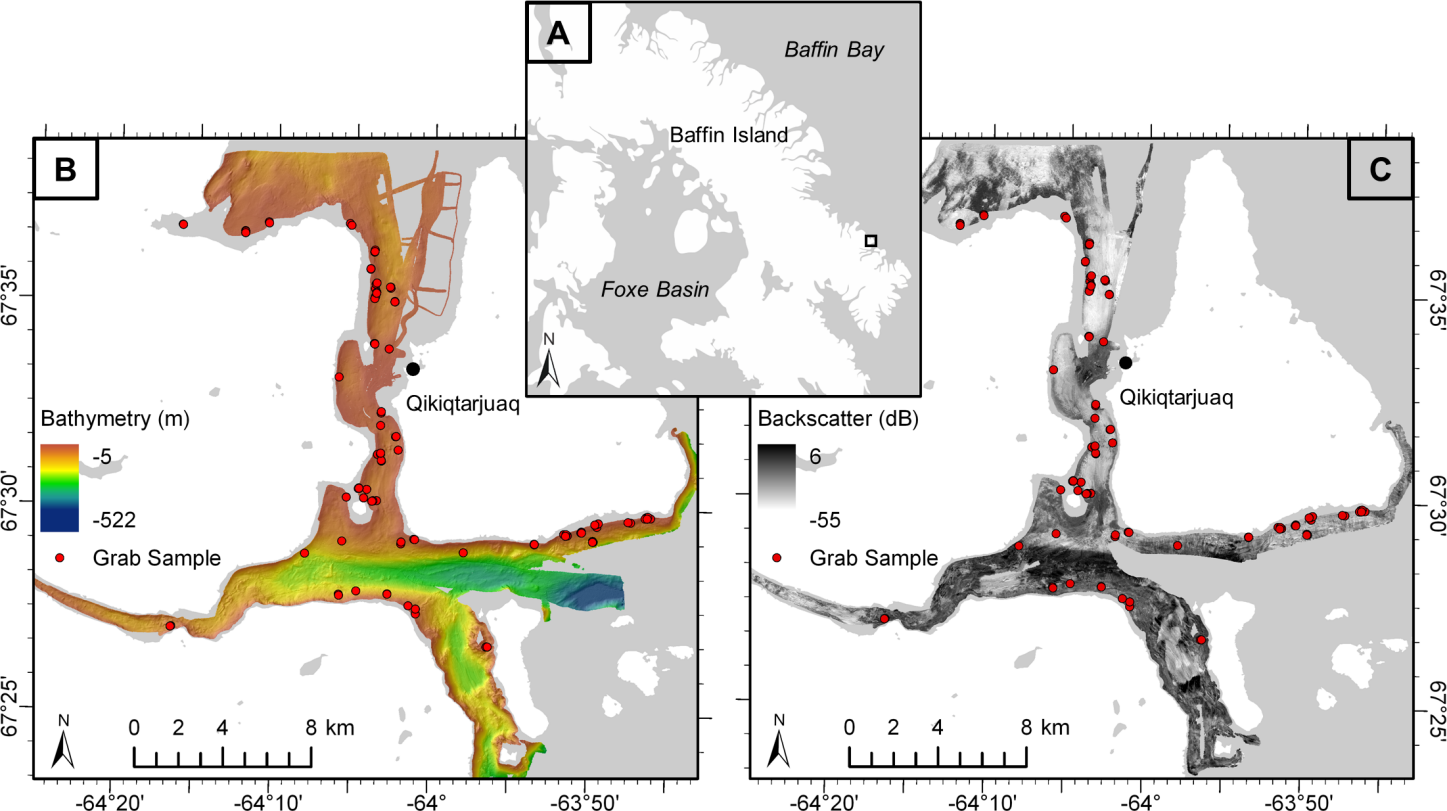
Study site. (A) Location of study site on east Baffin Island, NU, Canada. (B) Bathymetry data collected via MBES, with grab sample sites in red. (C) Backscatter data collected via MBES, with grab sample sites in red. (A) was modified from the USGS National Map, available under the public domain; basemap in (B) and (C) was obtained from the Canadian Land Cover GeoBase Series, containing information licensed under the Open Government Licence–Canada.

This study was conducted in the coastal zone near the hamlet of Qikiqtarjuaq on the east-central coast of Baffin Island, Nunavut, Canada (Fig 1A). The surrounding terrain is mountainous, hosting upland ice caps and alpine glaciers, shaped by repeated glacial cycles during the Quaternary [36]. For example, deep valleys and fjords (> 300 m deep, such as the one south of Qikiqtarjuaq; Fig 1B) channeled glacial ice flowing from inland source areas onto the continental shelf. Coarse-grained glacial deposits mantle the coastal terrain and extend offshore. The north-south channel is relatively shallow (60–70 m deep), and is shallowest opposite Qikiqtarjuaq (16 m deep), where a tombolo may have joined the two islands during the postglacial sea-level lowstand [37]. Surface currents of 0.5–0.8 m/s in the channel winnow the local seabed [38].

### Primary data

Bathymetry and backscatter data were collected using a MBES over the course of five years in the coastal zone near Qikiqtarjuaq (Fig 1). The surveyed region can be morphologically separated into two broad areas: 1) the relatively shallow channel, oriented north-south, separating Qikiqtarjuaq from Baffin Island; and 2) a deeper fjord basin oriented east-west located south of Qikiqtarjuaq (Fig 1B). The *CCGS Amundsen* collected data in the deepest area (> 600 m) using a Kongsberg EM300 30 kHz (variable beam width) echosounder in 2007 [39]. The Government of Nunavut scientific research vessel *RV Nuliajuk* collected MBES data using a Kongsberg EM3002 300 kHz (1.5° x 1.5° beam width) echosounder in 2012–2013 and a Kongsberg EM2040C 200–400 kHz (1° x 1° beam width) echosounder in 2014–2015 [40,41]. Datasets from the different survey years were harmonized and used as single, continuous layers for analyses. Details of how datasets from multiple MBES systems were harmonized are provided in supporting information (S2 File). Depths mapped by the *RV Nuliajuk* were between 5 and 350 m, and up to 522 m by the *Amundsen*. The mapped area was approximately 135 km^2^. Because the acoustic response of the seabed is dependent on MBES operating frequency, the 30 kHz *Amundsen* backscatter dataset, which differed substantially from the 300 and 200–400 kHz, was omitted, yielding an area of 112 km^2^ analyzed.

In addition to MBES data, 109 sediment grab samples were collected between 2014 and 2015 to measure the grain size of sediment (Fig 1B and 1C). Seabed sampling that impacted benthic fauna was permitted by Fisheries and Oceans Canada in 2014 and 2015 (license no. S-14/15-1041-NU and S-15/16-1010-NU-A1). Work in this region was conducted in collaboration with the Government of Nunavut, Department of Environment, Fisheries and Sealing Division in 2014, and was further permitted in 2015 by the Nunavut Research Institute (license no. 01 025 15N-M). Sample sites in 2014 were targeted to cover a previously completed shallow underwater image survey conducted between 0 and 40 m water depth. Data from this study were also appropriate for use in the current study. Sample sites in 2015 were selected randomly in the area of the MBES survey, stratified by bathymetry (up to 200 m depth), bathymetry-derived seabed slope, and backscatter, in order to obtain sediment samples at a range of these values. All sediment samples were collected using an 8.2 L Wildco® Ponar Grab.

### Secondary data

While many terrain attributes can be derived from bathymetric data to describe seabed morphology, Lecours et al. [42] recommended using a specific combination of six attributes that together capture most of the topographic structure of a surface. The Qikiqtarjuaq bathymetric data were used with the Terrain Attribute Selection for Spatial Ecology (TASSE) toolbox [43] in ESRI ArcGIS v10.3.1 to compute values for those six terrain attributes using a default 3 x 3 window of analysis. The six terrain attributes include eastness and northness (unitless sin- and cosine-transformed measures of orientation or aspect), relative difference to the mean value (RDMV; a unitless measure of topographic position), standard deviation (a measure of terrain variability; metres), slope angle (degrees), and local mean (metres water depth). Local mean was strongly correlated with the input bathymetry layer and thus was not included in further analyses.

In addition to the attributes identified by Lecours et al. [42], we derived a set of variables that may apply specifically to the distribution of sediment grain size. Seabed curvature (degrees per metre) influences current regimes and can be used to identify landform boundaries [44], while measures of relative seabed position such as benthic position index (BPI; metres) identify topographic highs and lows that can affect bottom currents and sediment transport [45,46]. Rugosity (the ratio of surface area to planar area) and the vector ruggedness index (the variability in surface orientation) are both unitless measures of terrain variability, which can describe seabed topography and substrate at appropriate scales (e.g. rough/rocky, flat/soft; [44]). Curvature measures were generated using the “Curvature” tool in ESRI ArcGIS v.10.3.1; BPI at broad and fine scales (scale factors of 100 and 250 metres, respectively; [47]), rugosity, and ruggedness were derived using the Benthic Terrain Modeler (BTM) toolbox [48]. Backscatter heterogeneity (hereafter Δbackscatter; dB), which is useful for differentiating coarse and fine substrates [49], was derived from the primary backscatter layer using the same function applied to calculate surface roughness (i.e., obtaining “minimum” and “maximum” 3 x 3 pixel neighborhood layers, then subtracting “maximum”—“minimum”; cf. “backscatter roughness”; [49]). ΔBackscatter was calculated using the “Focal Statistics” and “Raster Calculator” tools in ESRI ArcGIS v10.3.1. Last, distance from the coast (metres), a potential driver of grain size distribution [15], was calculated from a coastal polygon layer generated by Cowan [37] using Euclidean distance.

Data artefacts that were not visible in the bathymetry layer became apparent in some terrain attributes. These occurred most commonly at the interface between MBES datasets collected from different years, or near the depth limits of the MBES systems. Because terrain variable artefacts can affect habitat mapping results [50] we decided to exclude these areas from the analysis, resulting in several narrow data gaps (see supporting information; Fig D in S2 File).

All variables, except for distance from the coast (calculated independent of MBES data), were calculated at multiple scales by first deriving them from the original bathymetric and backscatter data at 5-m resolution then averaging them over increasing windows of analysis [26]. Variables were averaged over 3 x 3-, 5 x 5-, 9 x 9-, 13 x 13-, 21 x 21-, 35 x 35-, and 55 x 55-pixel neighborhoods using the “Focal Statistics” tool in ESRI ArcGIS v10.3.1. These neighborhoods followed the Fibonacci sequence (rounded up when even)—a convenient number series of increasing interval size [44,51]. This resulted in 129 potential variables for predicting the response of sediment grain size, at eight different spatial scales (Table 1).

**Table 1:** Multiple scale explanatory variables selected for modeling sediment grain size.

Variable		Scales (m)	Calculation Method	Method Source
Primary	Secondary			
Bathymetry		5,15,25,45,65,105,175,275	-	-
	Eastness	5,15,25,45,65,105,175,275	TASSE	[43]
	Northness	5,15,25,45,65,105,175,275	TASSE	[43]
	RDMV	5,15,25,45,65,105,175,275	TASSE	[43]
	Standard Deviation	5,15,25,45,65,105,175,275	TASSE	[43]
	Slope	5,15,25,45,65,105,175,275	TASSE	[43]
	Fine BPI*	5,15,25,45,65,105,175,275	BTM	[48]
	Broad BPI*	5,15,25,45,65,105,175,275	BTM	[48]
	Curvature	5,15,25,45,65,105,175,275	Curvature Tool	-
	Profile Curvature	5,15,25,45,65,105,175,275	Curvature Tool	-
	Plan Curvature	5,15,25,45,65,105,175,275	Curvature Tool	-
	Area	5,15,25,45,65,105,175,275	BTM	[48]
	Rugosity	5,15,25,45,65,105,175,275	BTM	[48]
	Ruggedness	5,15,25,45,65,105,175,275	BTM	[48]
Backscatter		5,15,25,45,65,105,175,275	-	-
	ΔBackscatter	5,15,25,45,65,105,175,275	Focal Statistics	[49]
Distance from Coast		-	Euclidean Distance	-

See text for explanation and discussion of individual variables and calculation methods.

*Fine scale BPI calculated with inner radius of 1 and outer radius of 20; broad scale BPI calculated with inner radius of 15 and outer radius of 50. Scale factors of 100 m (fine BPI) and 250 m (broad BPI) averaged over the increasing window sizes result in scales of 100, 300, 500, 900, 1300, 2100, 3500, and 5500 m for fine; 250, 750, 1250, 2250, 3250, 5250, 8750, and 13750 m for broad.

### Sediment grain size distribution modelling

#### Response variables

Ninety-eight grab samples were from locations within the MBES survey, and were used to model the distribution of sediment grain size. Following recommendations by Aitchison [52] and Stephens and Diesing [12], mud, sand, and gravel fractions were treated as compositional data that sum to 1 for each sample, allowing grain size classes to be considered concurrently. These data were transformed to an additive log-ratio (ALR) scale for use in modeling, resulting in values that are a ratio of two of the grain size classes, which can be back-transformed to yield predictions of mud, sand, and gravel after modeling [12]:

$${ALR}_{ms}=log(\frac{mud}{sand})$$

$${ALR}_{gs}=log(\frac{gravel}{sand})$$

Some sediment samples had mud or gravel fractions equal to zero, which may be due to one of the following: a) sample sites lacking sediment of a given size class; b) recovering amounts of a class that were too small to measure; or c) not retaining all size classes when sub-sampling sediment grabs (occurred occasionally in low-gravel areas). Since samples with zero values still provide valuable information on grain size composition, including those data points in the analysis was important. Since the log of zero is undefined, a replacement method was necessary for zero values to facilitate the grain size data transformation. The “simple replacement” method (reviewed in [53]), which replaces zero values with measurements less than the minimum recorded for a given size class, was used in this study. Following Lark et al. [54] and Diesing et al. [13], we replaced the observed zero values with values below the level of precision of our scientific equipment (1 x 10^−4^), maintaining the possibility that the size class did occur in trace amounts.

#### Statistical modeling

Boosted Regression Trees (BRT; [55,56]), a popular tree-based machine learning (ML) technique for ecological modeling [57], was applied to model the responses of ALR_ms_ and ALR_gs_ to the different explanatory variables at different scales (see Table 1). This non-parametric technique can accommodate large numbers of non-linear categorical and numerical explanatory variables simultaneously, while automatically modeling interaction between predictors [57,58]. BRTs were chosen because they commonly outperform other quantitative modeling methods [10,59–61], ignore unimportant variables, are insensitive to outliers, and tend to avoid over-fitting [55–57,59]. BRTs can provide plots of partial response, variable interaction, and measures of variable contribution, which allow the user to explore mechanistic relationships between explanatory and response variables [15,62]. Details on how BRTs work have been detailed by Friedman et al. [55], and with ecological examples by Elith et al. [62].

**Fig 2:**
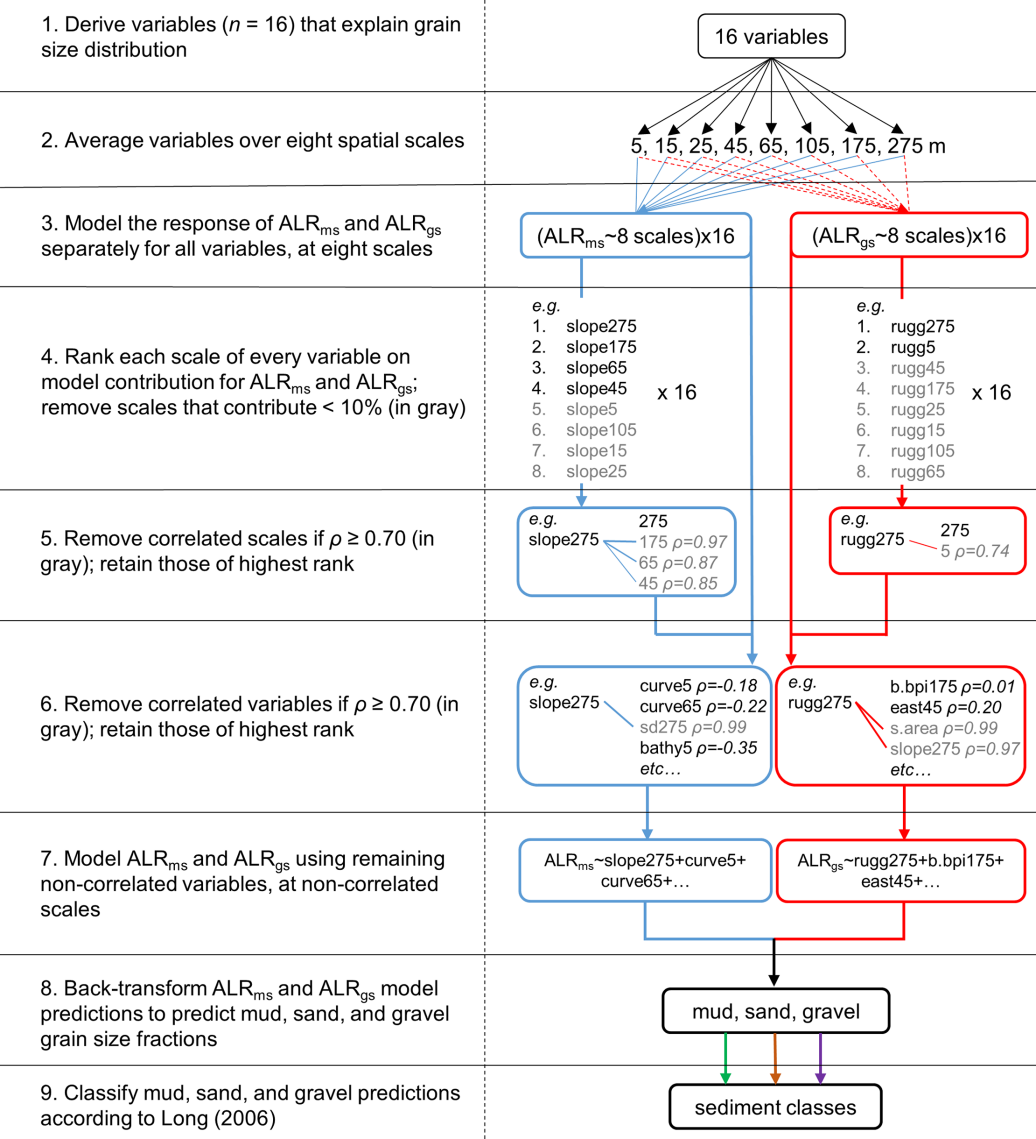
Sediment grain size modeling workflow. Procedure for selecting explanatory variables at multiple scales to model the response of ALR_ms_ and ALR_gs_ and predict the distribution of grain size classes.

While BRTs can accommodate large numbers of explanatory variables and tend to ignore those that are not important, it is still desirable to limit the number of variables in a model to facilitate understanding of the scale-dependent mechanisms that control grain size distribution [15,63]. BRTs can also be used as an exploratory tool to perform this function, as they return information on the importance of explanatory variables in predicting the response [62,64]. Thus, we first fitted models of both ALR_ms_ and ALR_gs_ iteratively for each individual variable, but at all scales (Fig 2, Step 4). These preliminary models provided two useful pieces of information: 1) a measure of which scales best explain the distribution of grain size for each variable, and 2) a ranked order of how well each variable, considered at multiple scales, explains the distribution of grain size. Percent relative importance was used to determine at which scales each variable performed best. Decision trees that comprised the BRT models were grown based on reducing the maximum amount of deviance in each model, thus models with the least residual deviance after fitting were considered best and were ranked as such. This methodology was applied separately for ALR_ms_ and ALR_gs_, resulting in two sets of both ranked variables and information on the best-performing scales for each variable. All modeling was conducted in R v3.2.3, with code modified from that provided by Elith and Leathwick [65] and Ridgeway [66].

Fitting individual models of ALR_ms_ and ALR_gs_ for all scales of a given variable provided information on the relative contribution of each scale, and only scales that contributed ≥ 10% to a given model were subsequently considered. Correlation between scales of a given variable was measured using Spearman’s rank correlation coefficient (Fig 2, Step 5). Correlated scales of a given variable were removed if *ρ* ≥ 0.7, giving preference to those that contributed more to a given model [22,67]. Once correlated scales of each variable were removed, correlation was assessed at all scales between different variables. Variables with correlation *ρ* ≥ 0.7 were removed, giving preference to variables that had the least residual deviance after model fitting (Fig 2, Step 6). This methodology allowed for the selection of the most important, non-correlated scales of each variable for inclusion in modeling ALR_ms_ and ALR_gs_. Models were then fitted for both ALR_ms_ and ALR_gs_ using the remaining non-correlated variables (Fig 2, Step 7).

ALR_ms_ and ALR_gs_ were back-transformed to produce predictions of mud, sand, and gravel fractions, individually (Fig 2, Step 8). Back-transformation was performed using the following functions in the “Raster Calculator” tool in ESRI ArcGIS v.10.3.1 [15]:

$$mud=\frac{Exp10({ALR}_{ms})}{Exp10({ALR}_{ms})+Exp10({ALR}_{gs})+1}$$

$$gravel=\frac{Exp10({ALR}_{gs})}{Exp10({ALR}_{ms})+Exp10({ALR}_{gs})+1}$$

sand=1−(mud+gravel)

These functions resulted in continuous predictions of mud, sand, and gravel fractions summing to 1 for all locations with MBES data. Following Stephens and Diesing [12], mud, sand, and gravel fractions were classified according to Long [68] to produce a single map of sediment distribution (Fig 2, Step 9). Long’s scheme combines these fractions into the classes “mud and sandy mud”, “sand and muddy sand”, “mixed sediment” and “coarse sediment”, allowing them to be represented simultaneously in a single map.

#### Model evaluation

Elith and Leathwick’s [65] extension to the Generalized Boosted Regression Models (gbm) package in R [66] implements an *n*-fold cross-validation (CV) procedure for BRT model building. CV partitions the response data into *n* folds, *n*-1 of which are used to train a model that is evaluated using the excluded partition. This is repeated *n* times, and the results are averaged to produce the final model and evaluation statistics. CV within Elith and Leathwick’s [65] code calculates average percent deviance explained over 10 model folds by default, which is useful for evaluating model fit [62,67]. An additional manual 10-fold CV was conducted to measure the average Spearman’s rank correlation between back-transformed predictions (i.e., mud, sand, and gravel fractions) and observed values of the withheld data partition. Spearman’s correlation coefficient provides a non-parametric ranked measure of monotone relationship between predictions and observed values, providing an indication of the model’s ability to predict grain size at new locations [69,70].

## Results

### Variable and scale selection

**Fig 3:**
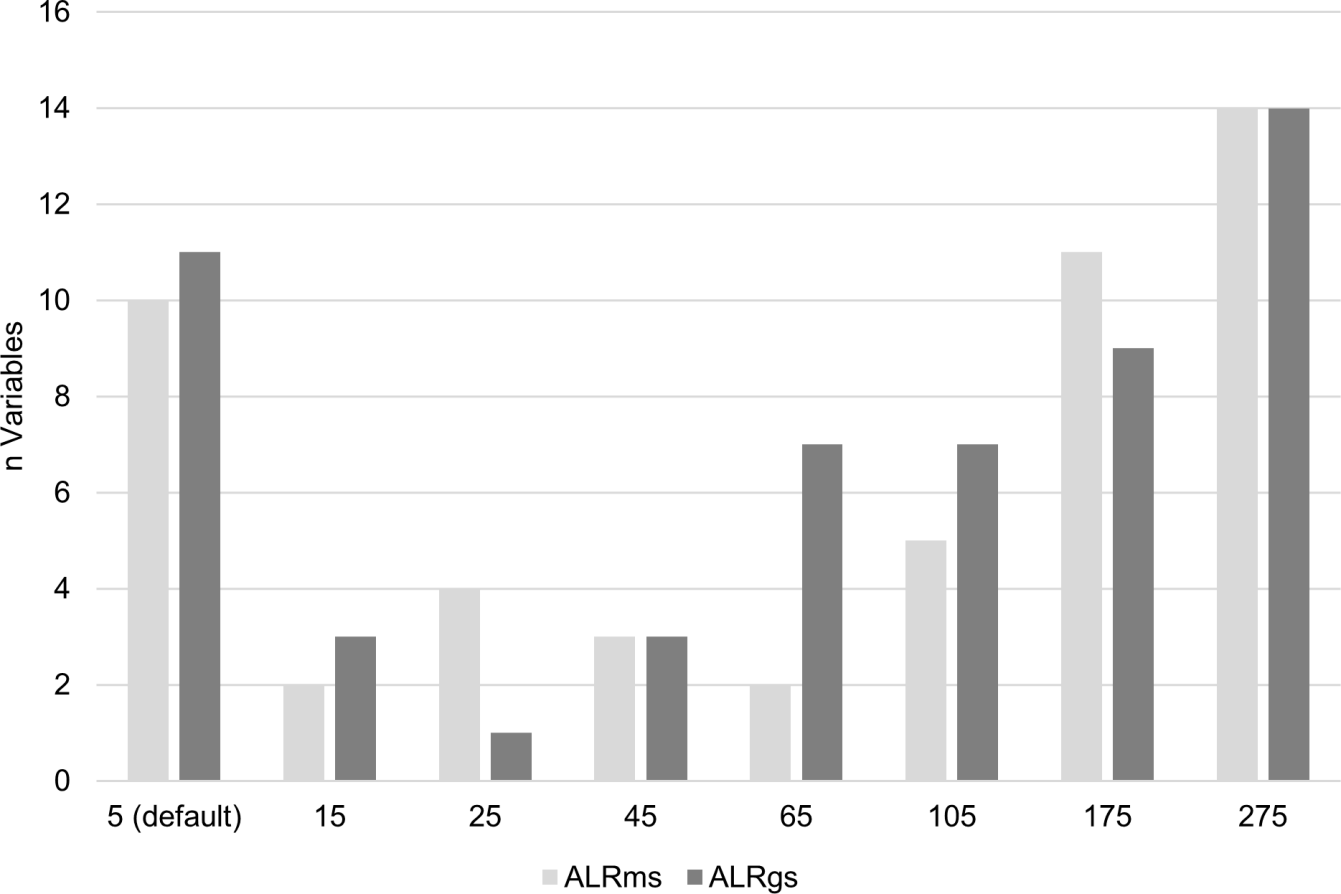
Scales selected for modeling. Number of times each scale contributed ≥ 10% to test models, and was selected for modeling.

ALR_ms_ and ALR_gs_ responses to the explanatory variables tested demonstrated that the default 5-m scale of analysis was not necessarily the most appropriate scale for all variables. Out of 51 scale-specific variables considered for modeling ALR_ms_ (i.e. those that contributed ≥ 10% to their respective single-variable model during testing) only 10 were at the default 5-m scale (Fig 3). Similarly, out of 55 scale-specific variables considered for modeling ALR_gs_, 11 were at the default 5-m scale. All scales tested were selected for modeling at least once, yet 5, 175, and 275 m were most common for both ALR_ms_ and ALR_gs_ (Fig 3).

**Fig 4:**
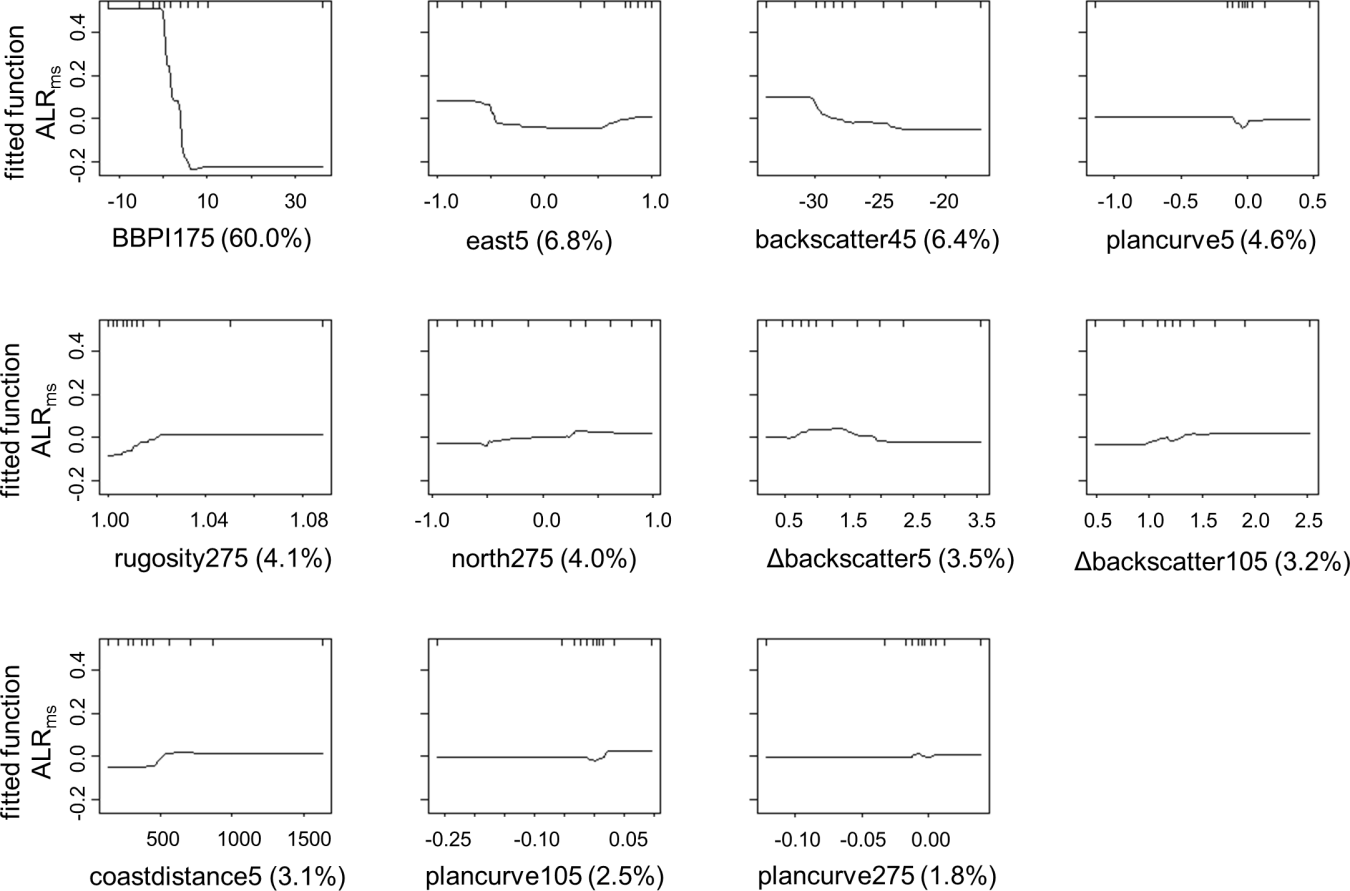
Variables selected to model ALR_ms_. Partial dependence plots for multiple scale variables selected to model ALR_ms_ with percent contribution to the model and data deciles on the upper x-axis.

Eleven variables were ultimately selected to model the response of ALR_ms_ (mud and sand) at five different scales (Fig 4): broad BPI (175 m), eastness (5 m), backscatter (45 m), plan curvature (5 m), rugosity (275 m), northness (275 m), Δbackscatter (5 m), Δbackscatter (105 m), distance from the coast, plan curvature (105 m), and plan curvature (275 m). ALR_ms_ was most strongly influenced by broad BPI at 175-m scale, the eastness component of aspect at 5-m scale, and backscatter at 45-m scale, together which contributed over 73% to model building. Partial dependence plots showed a strong negative trend between broad BPI and ALR_ms_. The ratio of mud to sand was generally higher on west-facing slopes and in areas of low backscatter response.

**Fig 5:**
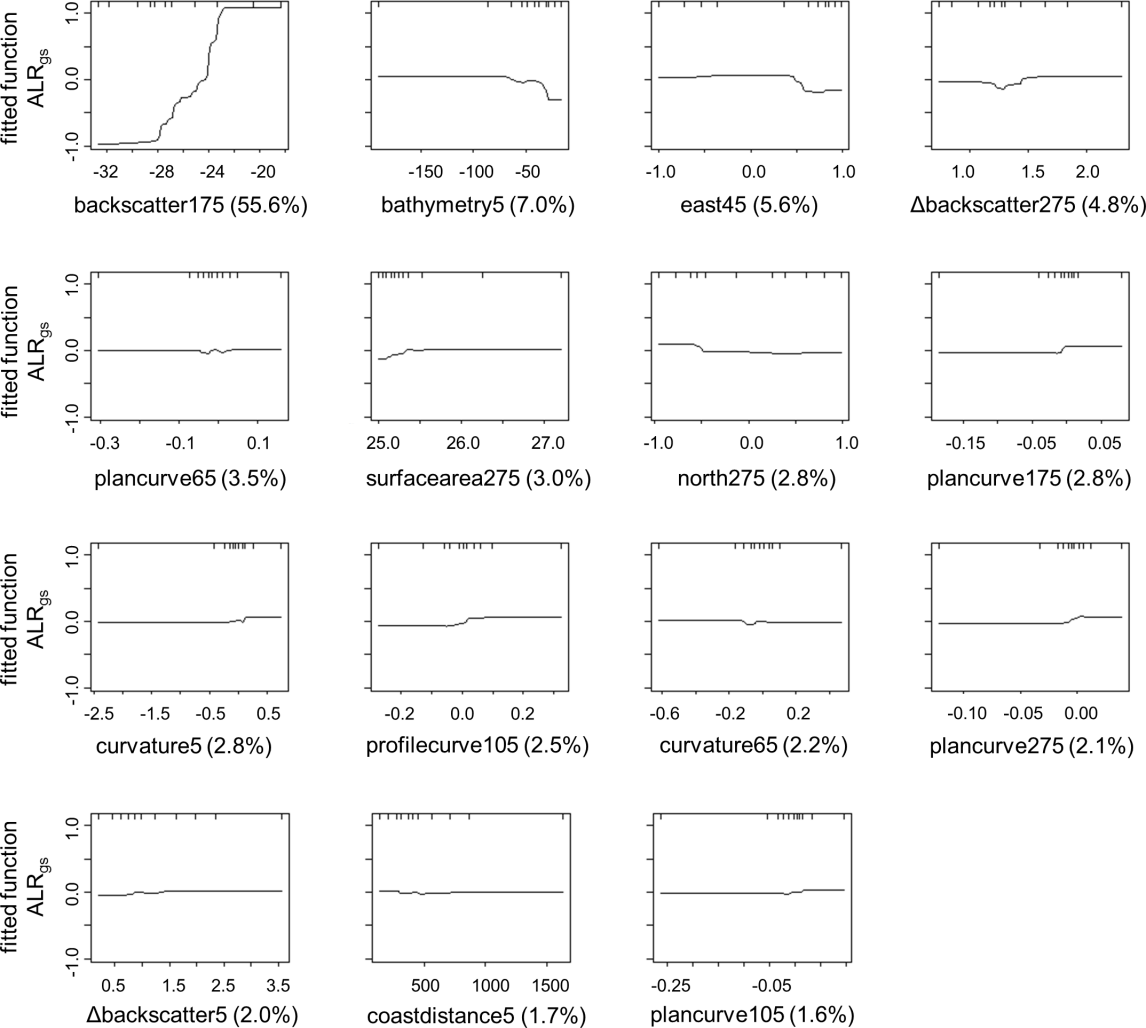
Variables selected to model ALR_gs_. Partial dependence plots for multiple scale variables selected to model ALR_gs_, with percent contribution to the model and data deciles on the upper x-axis.

Fifteen variables were selected to model ALR_gs_ (gravel and sand) at five different scales (Fig 5): backscatter (175 m), bathymetry (5 m), eastness (45 m), Δbackscatter (275 m), plan curvature (65 m), surface area (275 m), northness (275 m), plan curvature (175 m), curvature (5 m), profile curvature (105 m), curvature (65 m), plan curvature (275 m), Δbackscatter (5 m), distance from the coast, and plan curvature (105 m). ALR_gs_ was most strongly influenced by backscatter at 175-m scale, bathymetry at 5-m scale, and eastness at 45-m scale, together which contributed over 68% to model building. Partial dependence plots suggested a positive trend between backscatter response and ALR_gs_, a decrease of ALR_gs_ shallower than ~60 m depth, and a lower ALR_gs_ on east-facing slopes (Fig 5).

### Prediction

**Fig 6:**
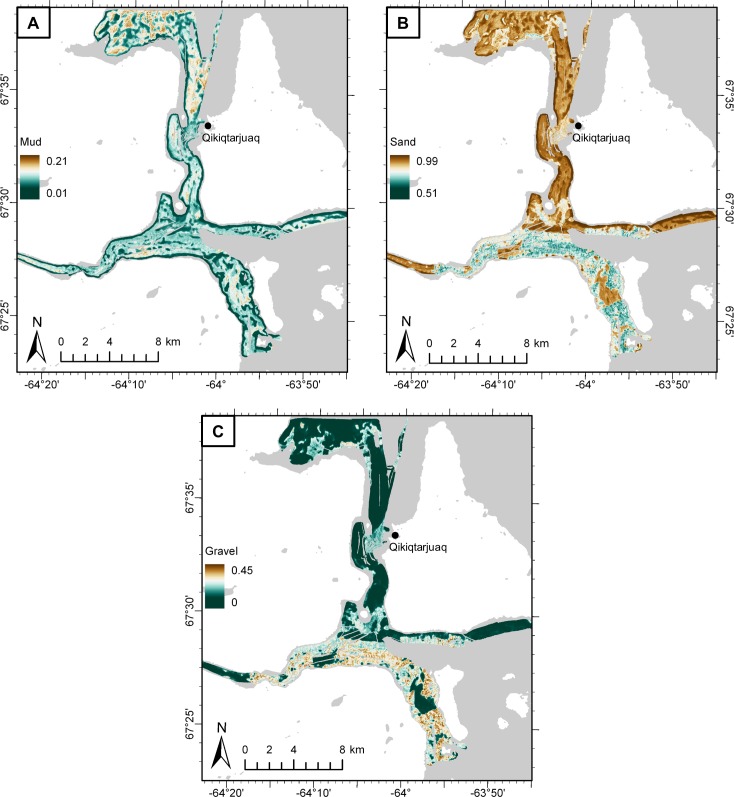
Potential grain size distribution of seabed substrate. Predicted proportions of A) mud, B) sand, and C) gravel fractions. Basemap from the Canadian Land Cover GeoBase Series, containing information licensed under the Open Government Licence–Canada.

Back-transformed additive log-ratios produced continuous predictions of mud, sand, and gravel fractions over the area of environmental data coverage (Fig 6). Sand was the dominant size fraction, comprising between 50.9 and 98.8% of sediment composition, with a mean of 82.3%. Sand was most abundant in the north-south oriented channel, and less so in the deeper fjord basin to the south (Fig 6B). Gravel comprised between 0 and 45.1% of sediment composition with a mean of 10.8% and was most abundant in the deepest waters to the south, where the east-west oriented fjord empties into Baffin Bay (Fig 6C). Mud was the least abundant size fraction, comprising between 0.8 and 21.6% of sediment composition with a mean of 6.9%. Mud was generally more abundant farther from shore, and was most common in patches north of the community of Qikiqtarjuaq (Fig 6A).

### Model evaluation

**Fig 7:**
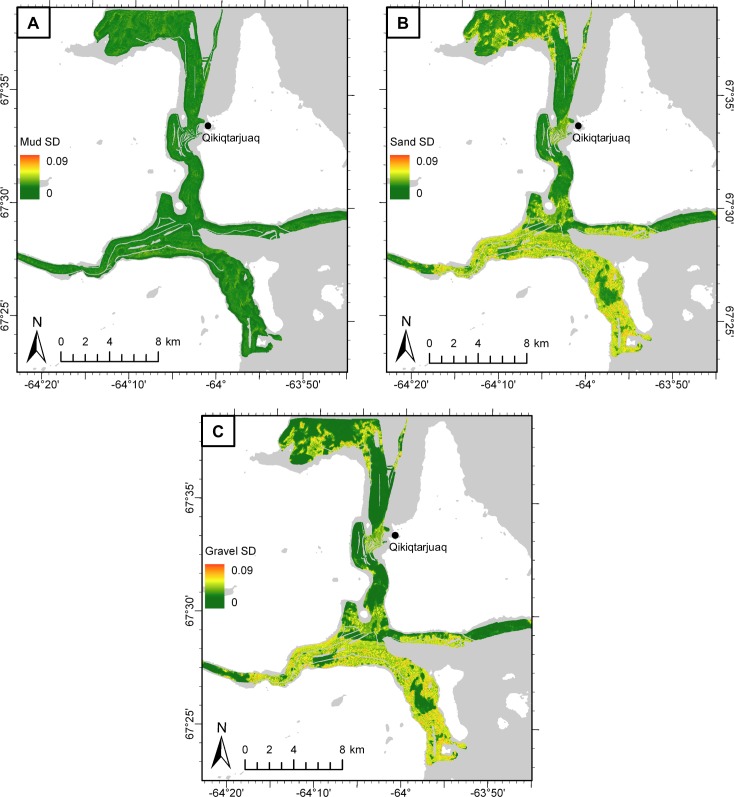
Grain size predictive uncertainty. Ten-fold CV standard deviations (SD) for A) mud, B) sand, and C) gravel predictions. Basemap from the Canadian Land Cover GeoBase Series, containing information licensed under the Open Government Licence–Canada.

On average over 10 folds, 56.3 and 46.4% of the statistical deviance was explained by the ALR_ms_ and ALR_gs_ models, respectively. Mud predictions had the highest average Spearman’s rank correlation (*ρ*_*mud*_ = 0.772), followed by sand (*ρ*_*sand*_ = 0.712) and gravel (*ρ*_*gravel*_ = 0.578). Ten-fold CV also produced a map of standard deviation for each grain size fraction, indicating areas of high and low model consensus (Fig 7). Mud predictions had the lowest mean standard deviation (*σ*_mud_ = 0.007), with the greatest model consensus in areas of low mud proportion, near the coasts (Fig 7A). Sand and gravel had similar mean standard deviations (*σ*_sand_ = 0.021, *σ*_gravel_ = 0.019) that were also distributed similarly in space (Fig 7B and 7C). Standard deviations for sand and gravel were highest in areas of high predicted gravel proportion and lowest in sandier areas.

### Classification

**Fig 8:**
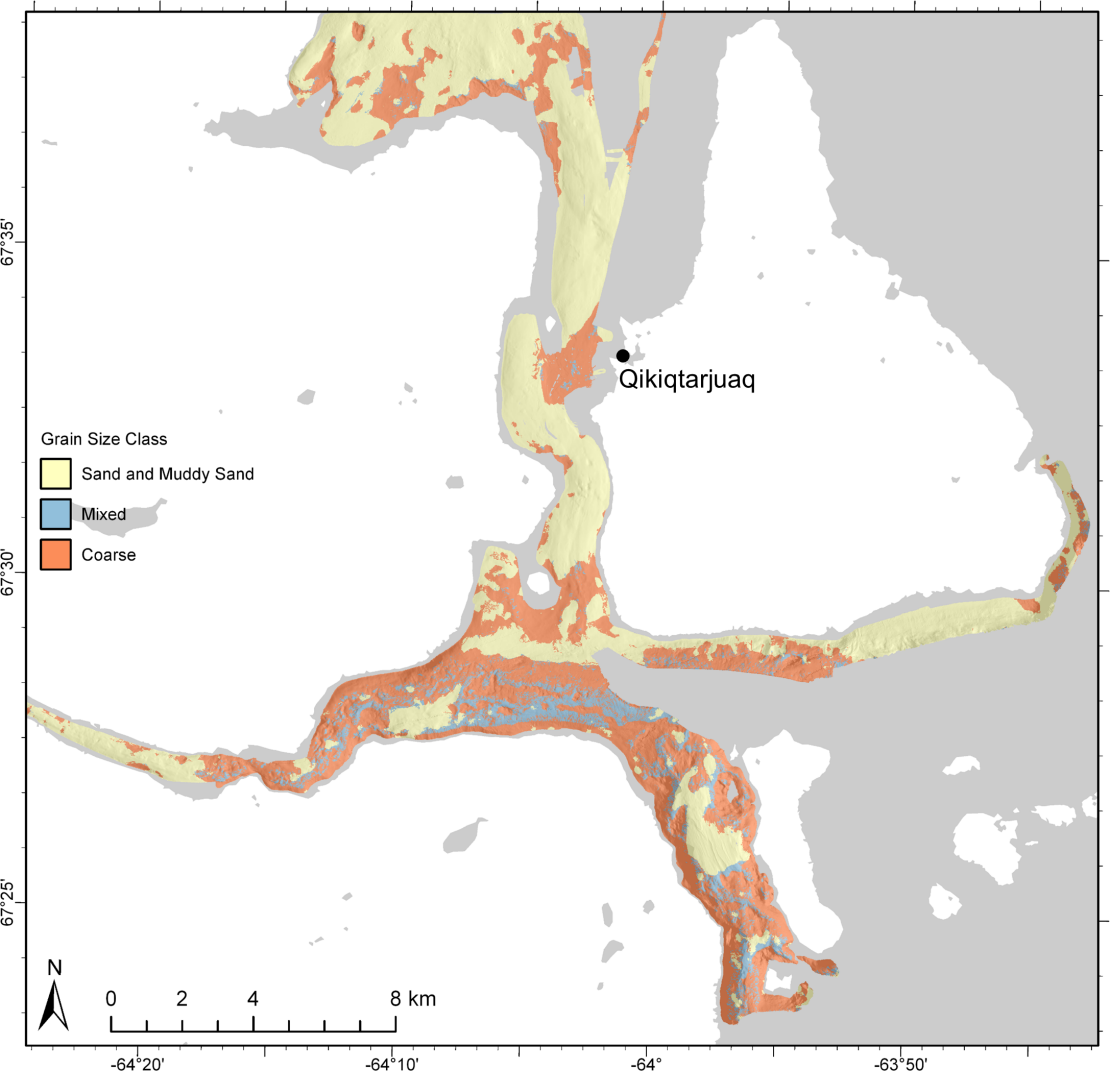
Grain size classification. Predictions of mud, sand, and gravel classified according to Long’s [68] modification of Folk’s [71] original classification scheme. See text for discussion. Basemap from the Canadian Land Cover GeoBase Series, containing information licensed under the Open Government Licence–Canada.

Following Stephens and Diesing [12], predictive maps of mud, sand, and gravel distribution were classified according to Long’s [68] modification of Folk’s [71] original scheme in order to facilitate interpretation and application as a management tool (Fig 8). This system was developed for use in the European Nature Information System (EUNIS) habitat classification, but we found it convenient for application to this study–it is simple and easy to interpret. A natural neighbor interpolation was applied to the individual grain size predictions in ESRI ArcGIS v.10.3.1 prior to classification to fill data gaps that were removed due to acoustic artefacts (see supporting information; Fig D in S2 File). The resulting map shows that the north-south oriented channel is composed primarily of “sand and muddy sand” except for the area proximal to Qikiqtarjuaq, which is “coarse”. “Sand and muddy sand” were predicted north of Qikiqtarjuaq with “coarse” patches at scales from 100s of metres to kilometres, with small patches of “mixed” sediment at scales from 10s to 100s of metres. “Sand and muddy sand” were predicted directly south of Qikiqtarjuaq, eventually coarsening farther south where the north-south oriented channel meets the east-west oriented fjord. “Coarse” patches in this area were predicted to occur over the scale of kilometres, with finer-scale patches of “mixed” sediment occurring over 10s to 100s of metres. “Coarse” and “mixed” substrates were predicted in the deep, high-relief, southernmost portion of the study area, with the exception of a ~3 x 1 km “sand and muddy sand”-filled basin in the middle of the channel. “Mud and sandy mud” occurred in such small quantities that the class was excluded from the map.

## Discussion

### Scale selection

The variable and scale selection process (Fig 2) demonstrated that the default scale of explanatory terrain variables was not necessarily the best option for modeling the distribution of sediment grain size, confirming previous observations that terrain variables are scale-dependent (e.g. [26,32,35]). Our methodology sought to identify the optimal scales at which explanatory variables influenced sediment grain size distributions. The default was sometimes identified as the best scale for an explanatory variable, but this was not always the case, and found to be not applicable for most variables. This has broader implications for management and habitat mapping efforts that use such modeling predictions. Since the use of the same environmental variables at different scales will ultimately produce different modeling results [44,45,64,72,73], the effects of scale selection will be propagated throughout the modelling process, and will impact the results of these efforts. For instance, ALR_gs_ models indicated that variables representing influences of bottom currents (e.g. aspect, curvature) were most appropriate at scales between 45 and 275 m (Fig 5). Variables were selected at these scales and used in the models to produce maps of grain size distribution. Map products would differ had they included these variables at the default resolution.

The broadest scales (175 and 275 m) were commonly selected for predictor variables, but fine and intermediate scales also contributed to the final models of ALR_ms_ and ALR_gs_. During step 6 of the modeling process (removing correlated variables; Fig 2) more than half of the variables selected at the coarsest resolution (275 m) were correlated with other variables, and were removed. Other authors (e.g. [45,74,75]) have noted the effect of “coarse-graining”, in which coarsening data resolution reduces the range of values, causing them to converge upon a mean. This effect may have caused increased correlation between broad scale variables in our study. Consequently, final models included fewer broad scale variables than were originally selected (Figs 4 and 5).

It is possible that importance of broad scale variables was not due entirely to their scale-dependent relationship to the response. Data coarsening can reduce the effects of ground truth locational inaccuracy, which was not quantified while grab sampling, but which could potentially affect model performance [67]. Noise present in the primary bathymetry and backscatter data layers can also be propagated, and even amplified in derivative layers [75], which can also affect model performance [50]. A decrease in spatial resolution can reduce data noise in derivative layers [46,67]. By averaging derivative data layers over an increasing area, noise can be smoothed out and made less distinct (see [32] for a comprehensive analysis of the effects of data coarsening on bathymetric derivatives). We found that data noise was less distinct at broader scales in this study. Though this may have produced a slight increase in the predictive ability of coarser scale data layers, the effect was not overwhelming—indicated by the frequent importance of fine scale variables identified during variable testing (Fig 3). A better understanding of the effects of error propagation in bathymetric data could clarify the impact of data coarsening on scale selection and model performance.

### Variable selection

Results from the variable selection process suggest that the morphology of the seabed strongly influenced ALR_ms_. The broad scale response of mud and sand to BPI (Fig 4) shows that the relative abundances of these size fractions were sensitive to topography over scales of 100s of metres to kilometres, with finer grain sizes increasing at topographic lows. During the variable and scale selection process, BPI was found to correlate with bathymetry (*ρ* = 0.79), yet the former was a stronger predictor of ALR_ms_ and was selected for the final model. This implies that broad BPI may have acted as a surrogate for bathymetry in the model, especially close to shore where BPI exhibited an edge effect caused by “no data” points outside the area of MBES coverage. This nearshore area of the BPI layer mimicked the shallowing of the bathymetry layer, which elicited a strong response in mud and sand predictions (Fig 6). Thus, we consider bathymetry to also be an important factor influencing the distribution of grain size, for which BPI was a surrogate. ALR_ms_ increased with distance from the coast (Fig 4), describing the increased transport of finer sediments.

Bottom currents transport sediments and control rates of erosion and deposition, making them one of the strongest drivers of sediment distribution [32,46,76]. Morphology influences the speed and orientation of currents, and also describes the exposure of seabed to them. Variables that describe seabed morphology, including bathymetry, eastness, northness, curvature, and slope, together can serve as surrogates for bottom currents. The importance of eastness at the 5-m scale in our study is potentially a result of its surrogacy for current information [34,77]. The moderate response of ALR_ms_ to backscatter at an intermediate scale (45 m) suggests that sand was slightly more acoustically reflective than mud (Fig 4). Backscatter information was most useful for the ALR_ms_ model when averaged over 45 m. MBES data collection was not optimized for backscatter data quality; averaging these data may have smoothed noise that was present in the data, which was impacting model performance [67]. It is possible that the selection of 45-m backscatter indicates a scale-dependence with ALR_ms_, yet, assuming backscatter is a proxy for substrate hardness, it is unclear why this relationship would be most apparent at the 45-m scale. Though slightly weaker predictors, plan curve and Δbackscatter variables had multiple non-correlated scales that contributed to the ALR_ms_ model. This suggests that these variables did not capture the same terrain information at different scales, and can be considered concurrently.

ALR_gs_ responded most strongly to backscatter averaged over 175 m, confirming that gravel was more acoustically reflective than sand (Fig 5). This corroborates findings by other authors (e.g. [78]) and highlights the usefulness of backscatter as a surrogate for bottom substrate properties (e.g. hardness, roughness)–it contributed over 55% of the information used to train the ALR_gs_ model. The broad scale relationship between backscatter and ALR_gs_ suggests that backscatter was a useful predictor of grain size averaged over a large area–or potentially for larger patches of sediment. This relationship also may have been affected by the noise reduction of the backscatter layer after averaging. The heterogeneity of backscatter over a broad area, represented by the Δbackscatter variable at 275-m scale, was useful for predicting ALR_gs_. A positive trend between these variables suggests that extensive gravelly areas caused increased variability in backscatter return, compared to sandy areas. Bathymetry and eastness were important predictor variables at fine scales, reinforcing the importance of high-resolution data in habitat mapping [7,34,45,79]. Measures of curvature (i.e., curvature, plan, profile) were weaker predictors of ALR_gs_, but were non-correlated at multiple scales, allowing each to be included in the model.

### Model prediction and evaluation

Sand was predicted to be the most abundant grain size fraction in the area, and gravel was predicted to occur in higher proportions than mud, but was less widespread across the study area (Fig 6). This is not surprising given the local igneous and metamorphic bedrock geology (granites and gneisses), which is scoured by glacial processes and overlain by sandy till veneer [80,81]. Although field observations and underwater video also suggested that sand was the dominant size fraction there is the possibility of sampling bias, which may have influenced these results. Generally, grab sampling was most successful in sandy areas. Compact muddy sediments and gravel both occasionally limited the penetration of the grab sampler, and high-gravel proportions were typically not captured in the grab, or caused sample loss. For instance, the field team noted some areas composed almost entirely of clasts ranging from pebbles to cobbles, yet the lowest predicted proportion of sand was 0.51. Fewer successful grab samples in gravelly areas may have contributed to its lower predicted abundance compared to sand. Despite the potential for bias, these results seem to accurately represent most of the study site. For instance, grab sampling, underwater video, and field observation all suggested that the north-south oriented channel was primarily sand, with little mud or gravel, except near Qikiqtarjuaq (Fig 8).

Percent deviance explained, calculated internally using withheld data over the 10 CV model folds for ALR_ms_ and ALR_gs_, provided a measure of quality for model fit (i.e., calibration [62,82,83]), and Spearman’s correlation coefficient calculated for predictions of mud, sand, and gravel indicated the model’s ability to quantitatively predict grain size fractions in un-sampled locations (i.e., discrimination [83]). Each modeling scenario is different, and there is no objective threshold of percent deviance explained at which a model is considered “well-calibrated”. Regardless, the percent deviance explained in our models of ALR_ms_ and ALR_gs_ on withheld CV data (56.3% and 46.4%, respectively) compare favorably with the literature (e.g. [67,83,84]). This metric was also useful in the variable and scale selection process because it provided a relative indication of goodness-of-fit, allowing for comparison between prospective models (i.e. Fig 2, step 4). Spearman’s correlation coefficient for 10-fold CV predictions of mud (*ρ*_*mud*_ = 0.772), sand (*ρ*_*sand*_ = 0.712), and gravel (*ρ*_*gravel*_ = 0.578) indicated a strong positive association between predicted and observed grain size fractions. Gravel had the lowest correlation score, with high-gravel areas (Fig 6C) displaying the most variability between the 10 model folds, as measured by standard deviation (Fig 7). We suspect that the difficulty in predicting the gravel fraction was largely due to the bias in grab sampling.

**Fig 9:**
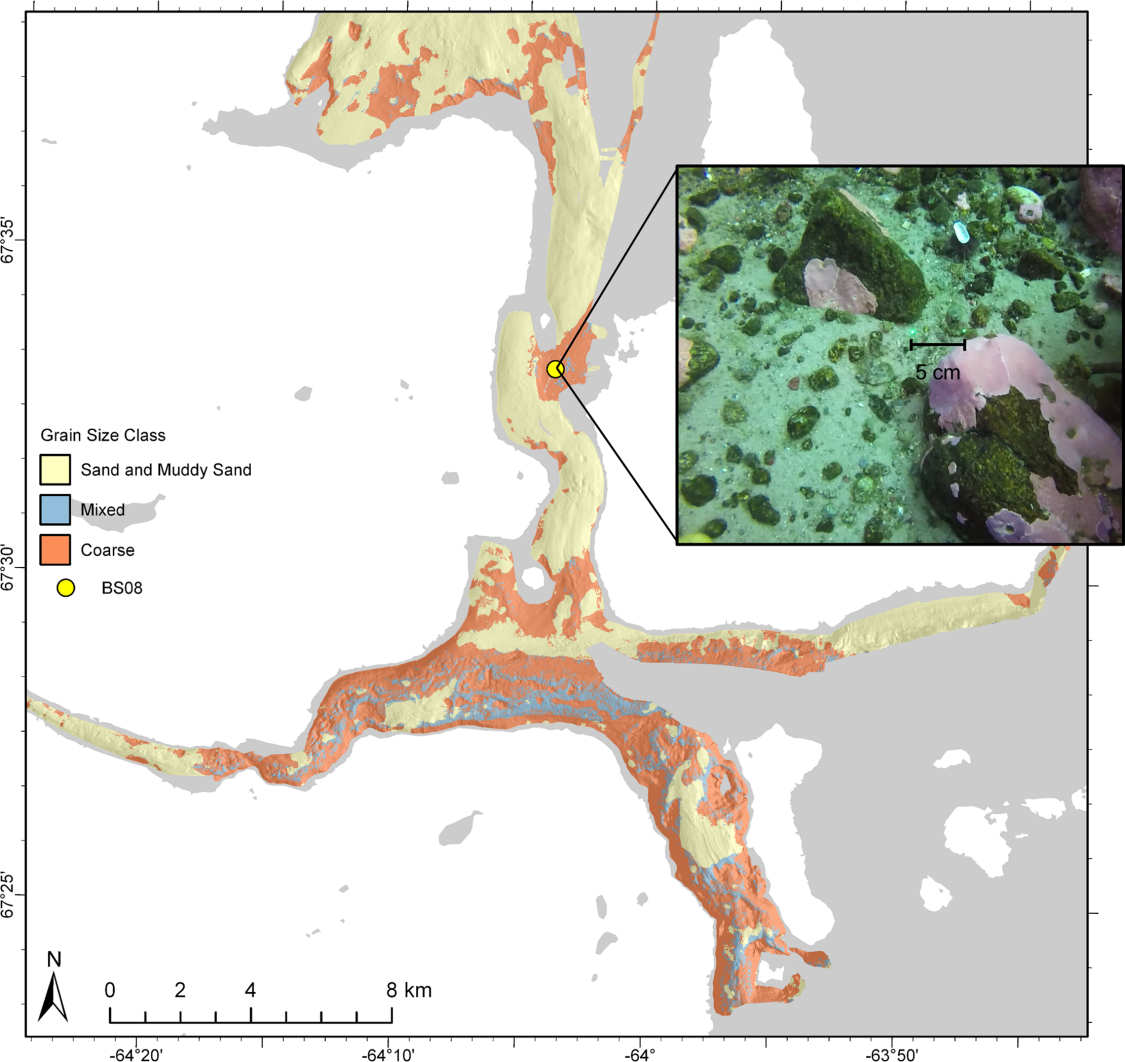
Large pebble and cobble observation. Clasts too large to sample in an area classified as “coarse”, with 5-cm scale lasers. Basemap from the Canadian Land Cover GeoBase Series, containing information licensed under the Open Government Licence–Canada.

Maximum grain size retained was also a limiting factor to this study. The field team noted difficulties in retaining sediment grains > 4000 μm, effectively limiting the ability to model sediments larger than small pebbles. This means that large gravel was not predicted. Thus, the map of the gravel fraction (Fig 6C) represents the distribution of gravel ≤ 4000 μm, and the “mixed” and “coarse” classes (Fig 8) only include substrates up to this size. For instance, the presence of cobbles was obvious in the area near Qikiqtarjuaq from underwater video and field observations, yet model outputs were simply classified as “coarse” (Fig 9), which represents the substrate component surrounding larger clasts. These predictions are valid and useful from an ecological perspective, but it is important to understand their limitations. Future work could investigate methods for integrating larger clasts observed in underwater video with sediment grain size predictions (e.g. [85]).

Sampling was limited to 200 m depth by equipment performance, making validation of predictions in the deepest parts of the study area impossible. For example, high backscatter response in the deep east-west oriented fjord (Fig 1C) suggests a coarser grain size, yet these predictions could not be validated. Lack of sample sites in this area likely contributed to a higher standard deviation between model predictions (Fig 7). Despite this limitation, manual inspection suggests that predictions were largely based on high backscatter return (Fig 1C). Backscatter was the single most important variable in predicting ALR_gs_, accounting for over 55% of explained deviance in the model (Fig 5), providing some confidence that these predictions are well-founded.

### Classification

Long’s [68] simplification of Folk’s [71] scheme was chosen to classify sediment grain size predictions because of its generality. The objective in classifying grain size predictions was to create an accessible resource for scientists and managers, yet Folk’s classification, which places grain size into one of 15 categories, is complex and less accessible to non-experts. Long’s modification places samples into one of only four groups, which uses simpler terminology (e.g., “gravelly muddy sand”, “muddy sandy gravel”, “muddy gravel”, and “gravelly mud” are grouped into the class “mixed”). Each of the four classes occurred in this study area, but the class “mud and sandy mud” was very rare, and was ultimately excluded because it did not add meaningful information to the map (Fig 8). The quantitative predictions of mud, sand, and gravel produced in this study can be readily classified into any other sediment grain size scheme based on user need [15].

## Conclusions

Results of this study demonstrate that the default data resolution of each terrain variable was not necessarily at the appropriate scale for explaining the distribution of grain sizes for seabed sediment. Terrain variables acting as surrogates for seabed morphology and hydrodynamics (e.g. BPI, bathymetry, and aspect), implemented at broad and fine scales, were the most important variables for differentiating the mud and sand fractions. Broad scale backscatter was the most important variable for distinguishing gravel from sand; terrain variables were of secondary importance. Multiple scale models were used to predict the distribution of the different sediment grain sizes, avoiding the arbitrary selection of spatial scale for explanatory variables. The results of this analysis can be used quantitatively in subsequent habitat mapping studies, or can easily be reclassified based on management need.

These findings highlight the importance of considering variables at multiple scales for seabed mapping. By failing to test for scale-dependence of explanatory variables in predicting the response we risk creating less realistic maps. Multiscale and multiple scale analyses should not be considered a specialized form of analysis. We recommend that scale be considered an integral part of any benthic habitat mapping procedure–at least as important as variable selection.

Future work on the difference between mapping products as a result of multiscale derivation method (see [26]) could elucidate the importance of choosing one derivation method over another. Though there is strong evidence that different methods of deriving variables at multiple scales produce different products, it is not clear how the end products of a study may differ based on multiscale method.

## Supporting information

S1 File Sediment grain size data. Mud, sand, and gravel size fraction data for grab samples collected near Qikiqtarjuaq, NU.
(XLSX)

S2 File MBES data harmonization methodology. Detailed description of methods used to combine MBES data from multiple survey years and systems.
(PDF)
