# Supplementary material for: A multiscale approach to mapping seabed sediments
Source: PLoS One. 2018 Feb 28;13(2):e0193647. doi: 10.1371/journal.pone.0193647 (PMC5831638; doi:10.1371/journal.pone.0193647)
Supplement: S2 File — Detailed description of methods used to combine MBES data from multiple survey years and systems. (PDF) [file pone.0193647.s002.pdf]

Backscatter data harmonization

The use of multiple non-calibrated backscatter datasets presents several difficulties, yet due to the high cost of data collection and the importance of these data for seabed mapping, methods have been developed to facilitate their combination. Seabed acoustic reflectivity has been used as a proxy for bottom hardness and substrate properties, yet acoustic measurements are dependent on several water column conditions (e.g. temperature, salinity), MBES system-specific parameters (e.g. operating frequency, pulse length), and survey conditions (e.g. vessel speed, survey overlap) [1]. For measurements to represent seabed characteristics, radiometric and geometric corrections must be applied to raw backscatter intensity to correct for these factors [1]. Corrections to raw backscatter intensity produce relative measurements that are specific to a single MBES system, and often to a single survey, unless calibrations have been performed to the systems prior to the survey. Furthermore, even after confounding factors have been controlled for, relative backscatter measurements are a function of operating frequency as well as substrate properties [2,3].

There are two broad methodologies for combining non-calibrated backscatter datasets: analyzing datasets separately and combining results *post-hoc* (e.g. [1]), or combining them into one harmonized dataset prior to analysis (e.g. [2]). We tested both methodologies for our data, and found that modelling the response of grain size to the single harmonized backscatter dataset resulted in maps with less-noticeable boundaries and edge effects caused by dataset combination (Fig A). The harmonized approach also produced more accurate results than creating independent models of each backscatter dataset and combining the results (Table A).

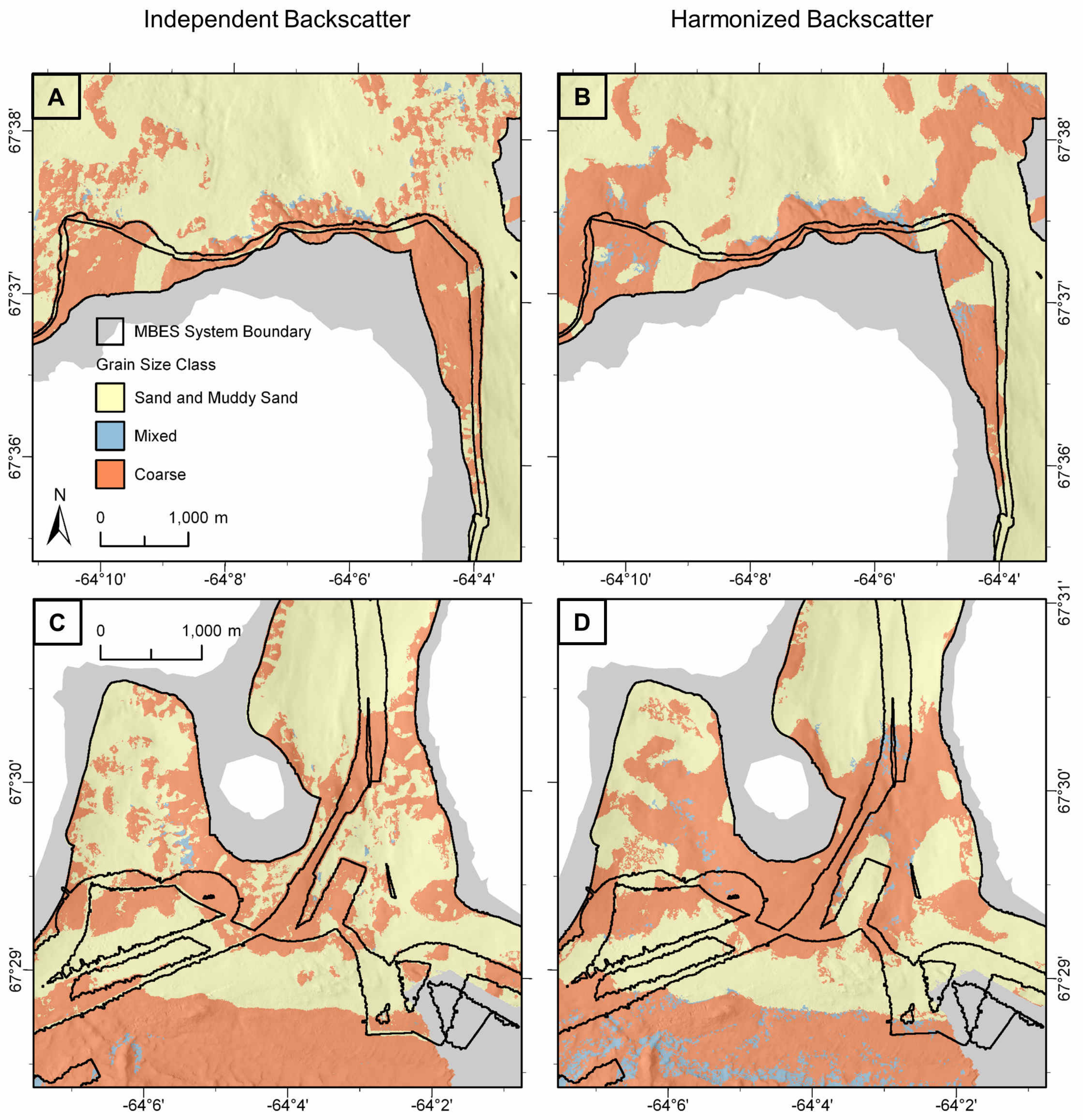

Figure A. Grain size class comparison. Differing grain size class predictions from modelling independent (A, C), and harmonized (B, D) backscatter datasets**.** Basemap from the Canadian Land Cover GeoBase Series, containing information licensed under the Open Government Licence – Canada.

Table A. Accuracies of independently-modelled backscatter mosaics and harmonized backscatter mosaic.

	ρ_{mud}	ρ_{sand}	ρ_{gravel}	**ALR_{ms} Deviance Explained**	**ALR_{gs} Deviance Explained**
**EM3002**	0.744	0.524	0.439	44.8%	17.4%
**EM2040C**	0.331	0.677	0.500	44.1%	59.2%
**Average of Independent Models**	0.538	0.600	0.469	44.4%	38.3%
**Harmonized**	0.772	0.712	0.578	56.3%	46.4%

Similar to [2] we applied bulk shifts to align backscatter datasets from different surveys to create a single harmonized backscatter dataset. Using the most extensive survey as reference, we observed how the surveys differed in areas where they overlapped. Hughes Clarke et al. [2] applied bulk shifts to backscatter datasets from five different MBES systems ranging from 93 to 300 kHz by adding or substracting the relative differences (in dB) of overlapping survey areas with respect to a reference backscatter dataset. We attempted both this method and a bulk shift that multiplied the backscatter datasets from different surveys based on the median factor by which datasets differed with respect to the reference (EM3002 in 2012). The multiplication method affected the range of the data as well as the median, and produced backscatter layers that were closer to the reference on average, with a lower standard deviation (Table B). Visual analysis suggested that this method was effective at harmonizing backscatter datasets from different surveys and MBES systems (Fig B), with few noticeable boundaries between the different datasets in the resulting layer.

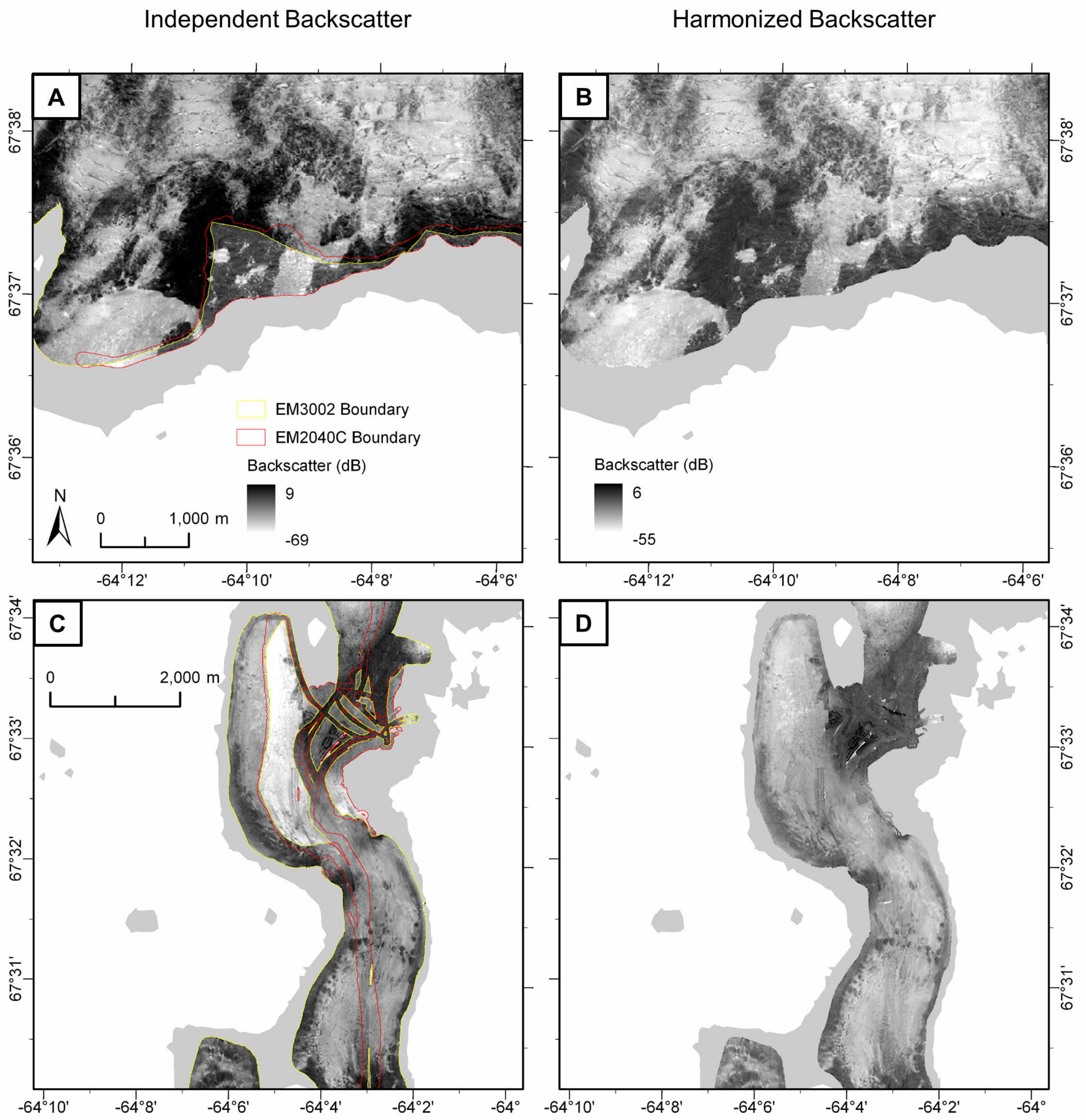

Figure B. Backscatter dataset comparison. (A, C) Non-corrected backscatter mosaics, and (B, D) bulk shift harmonized backscatter mosaic. Basemap from the Canadian Land Cover GeoBase Series, containing information licensed under the Open Government Licence – Canada.

Table B. Average absolute differences between independent backscatter mosaics with respect to reference survey (2012; EM3002) after bulk shift.

	2013 (EM3002)		2014 (EM2040C)		2015 (EM2040C)	
	Additive Shift	Factor Shift	Additive Shift	Factor Shift	Additive Shift	Factor Shift
Mean (dB)	2.17	2.16	2.67	2.59	3.03	2.75
SD (dB)	1.99	1.96	2.41	2.32	2.84	2.56

Our decision to use a single harmonized backscatter dataset that integrates surveys from four different years was based on the performance of this dataset compared to using them individually, and the quality of the map products. We acknowledge that the harmonized backscatter layer is a relative and imperfect proxy for seabed hardness, yet we believe this is the most appropriate way to utilize such valuable data. Furthermore, the harmonized backscatter layer was the single most important variable for differentiating gravel from sand; the boosted regression tree algorithm that we used for modelling ignores noisy or non-important variables [4], suggesting that the harmonized backscatter data produced using our methods was useful as a proxy for substrate properties.

Bathymetry and terrain variable harmonization

Bathymetric raster layers from each survey were mosaicked to a 5-m grid to form a single harmonized layer prior to deriving terrain variables and averaging to create multiple scales. The beam width of the EM3002 echosounder was 1.5° x 1.5°, the EM2040C was 1° x 1°, and the EM300 (backscatter not used) was variable between 1° x 1°, 1° x 2°, 2° x 2°, and 2° x 4°, meaning that the acoustic footprint and sounding density differed between the systems. The differences in inherent data resolution of the MBES systems may have resulted in small discrepancies in bathymetric resolution when gridded as a 5-m raster, which

were not initially apparent, but which manifest in derivative variables [5]. We applied a low-pass smoothing filter to high order variables that seemed potentially affected by the differences in inherent resolution (e.g. Fig C), including measures of curvature and relative difference to the mean value (RDMV; a measure of topographic variability). This issue is also resolved when raster layers are averaged to create coarser-scale variables, which is a positive byproduct of a multiple scale approach. The performance of these variables and others at fine scales (Figs 3-6 in manuscript) suggested that the low-pass filter was effective at reconciling differences in inherent resolution, and that discrepancies between data from the different echosounders did not seriously damage the use of these variables as predictors.

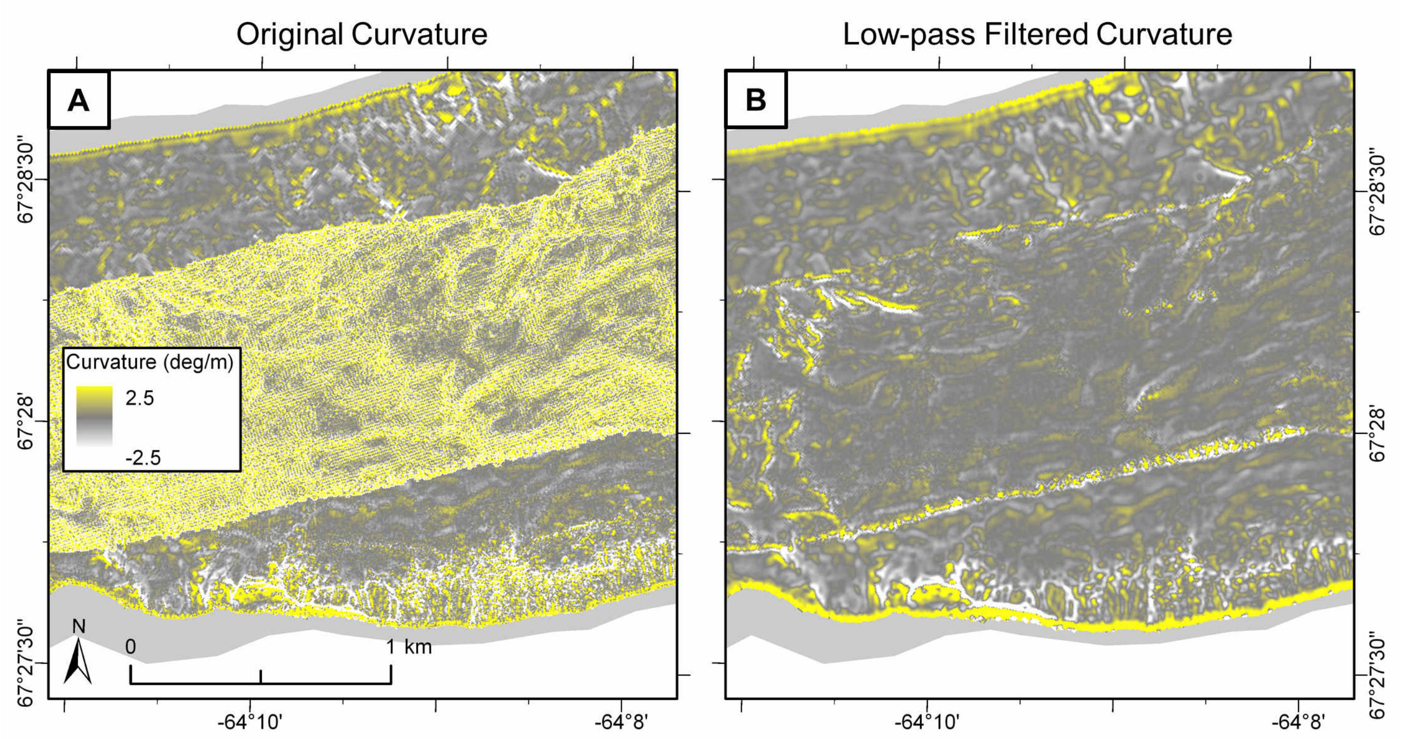

Figure C. Low-pass filtering of data with different inherent resolutions. (A) Curvature layer derived from 5-m gridded bathymetry data. (B) Curvature layer after low-pass filtering. Basemap from the Canadian Land Cover GeoBase Series, containing information licensed under the Open Government Licence – Canada.

Even after applying a low-pass filter to the variables listed above, there were clear artefacts present in some areas where surveys from different years overlapped (Fig C). Rather than risk impacting the analysis with these incorrect measurements, we elected to remove them prior to modelling (e.g. Fig D). These areas were omitted from all variables in order to maintain equal geographic extent.

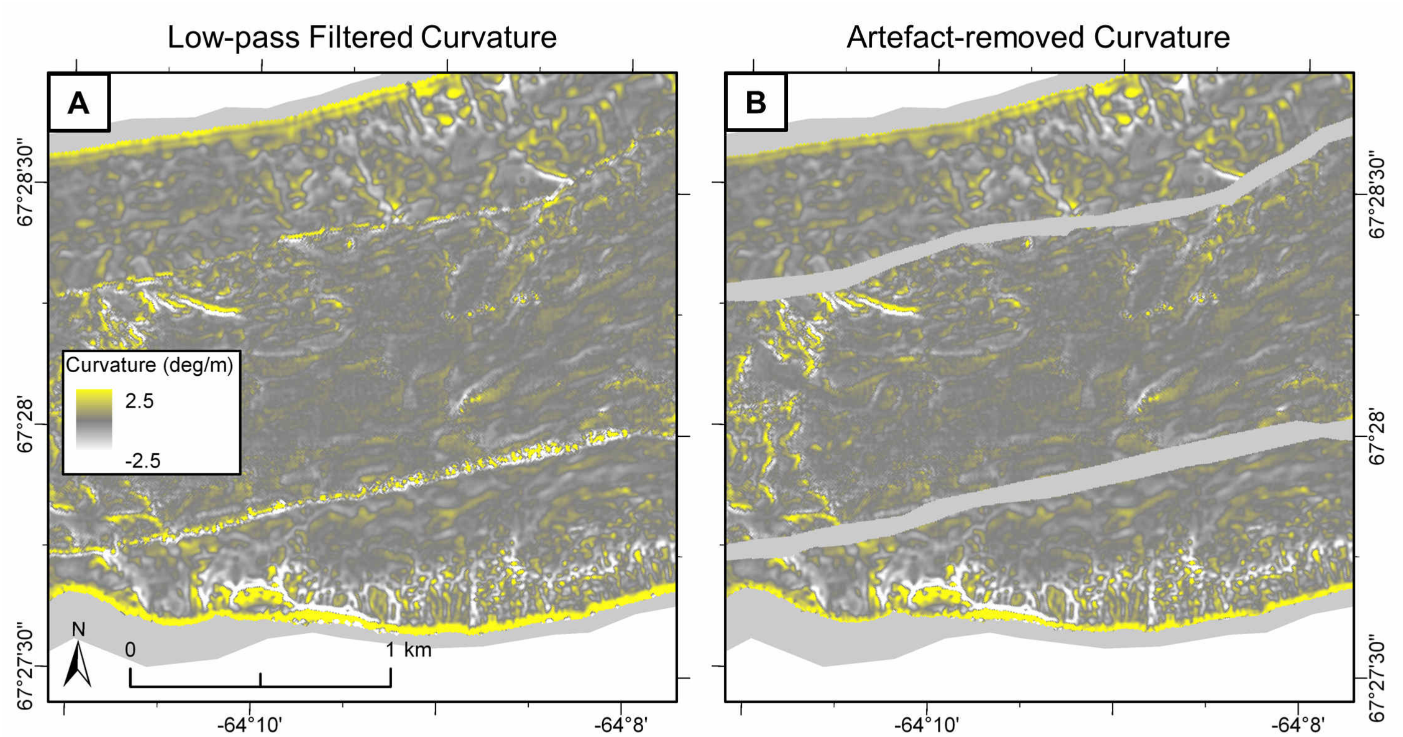

Figure D. Removing dataset overlap artefacts. (A) Curvature layer after low-pass filtering. (B) Curvature layer after removing artefacts at MBES survey boundaries. Basemap from the Canadian Land Cover GeoBase Series, containing information licensed under the Open Government Licence – Canada.

References

1. Lacharité M, Brown CJ, Gazzola V. Multisource multibeam backscatter data: Developing a strategy for the production of benthic habitat maps using semi-automated seafloor classification methods. Mar Geophys Res. 2017. doi: 10.1007/s11001-017-9331-6.

2. Hughes Clarke JE, Iwanowska KK, Parrott R, Duffy G, Lamplugh M, Griffin J. Inter-calibrating multi-source, multi-platform backscatter data sets to assist in compiling regional sediment type maps: Bay of Fundy. Proceedings of the Canadian Hydrographic Conference and National Surveyors Conference; 2008; Victoria, BC; paper 8-2.

3. Hillman JIT, Lamarche G, Pallentin A, Pecher IA, Gorman AR, von Deimling. Validation of automated supervised segmentation of multibeam backscatter data from the Chatham Rise, New Zealand. Mar Geophys Res. 2017. doi: 10.1007/s11001-016-9297-9.

4. Elith J, Leathwick JR, Hastie T. A working guide to boosted regression trees. J Anim Ecol. 2008;77: 802-813.

5. Hughes Clarke JE. Dynamic motion residuals in swath sonar data: Ironing out the creases. The International Hydrographic Review. 2003 Jan 1;4(1): 6-23.
